# Assessment of Biolog Ecoplate^TM^ method for functional metabolic diversity of aerotolerant pig fecal microbiota

**DOI:** 10.1007/s00253-021-11449-x

**Published:** 2021-07-23

**Authors:** A. Checcucci, D. Luise, M. Modesto, F. Correa, P. Bosi, P. Mattarelli, Paolo Trevisi

**Affiliations:** Department of Agricultural and Food Sciences, University of Bologna, 40127 Bologna, Italy

**Keywords:** Pigs’ microbiota, Functional diversity, Metabolism, Biolog^TM^ Ecoplates, Sample’s conservation

## Abstract

**Abstract:**

In the last decades, gut microbiota and its role in mammal host development and health have been increasingly investigated. Metabolites produced by gut microbiota can affect intestinal homeostasis and immune system maturity and activation, and in turn, they can influence the health and growth performance of livestock. Therefore, a better understanding of the functional metabolic capability of the gut microbiota would be appreciated by the scientific community. In this study, the Biolog^TM^ Ecoplates technology was applied for studying the metabolic potential of the aerotolerant microbial community of pig fecal samples, evaluating the interference of different storage conditions and cell concentrations. The length of time for which a fecal sample maintained detectable and unchanged microbial metabolic activity was also investigated. Two assays aimed to evaluate differences in the metabolic activities between fresh and snap-frozen fecal samples at different dilutions and at different lengths of times of preservation at −80°C were carried out. The biodiversity and the predicted functionality of the entire bacterial community through a targeted metagenomic approach were also explored. The results highlighted that snap freezing of fecal samples preserved the metabolic activity of the microbial community when compared to fresh feces. Sample storage at −80 °C did not significantly affect the metabolic activity of the microbial community, which was stable for 150 days. Furthermore, the highest metabolic activity was detected with 1:2 to 1:5 dilutions of the stock suspension. Biolog^TM^ Ecoplates technology is a rapid and useful method to explore microbial communities’ metabolism in animal fecal samples contributing to investigate host animal physiology.

**Key points:**

• *Freezing of samples can preserve the functional activity of the aerotolerant microbial community for 150 days.*

• *The concentration of microbial cells strongly influences metabolic activity detection.*

• *Sequencing coupled with the Biolog*^*TM*^ *Ecoplates could be a strategy to evaluate the metabolic potential of the microbiota of the fecal sample.*

**Graphical abstract:**

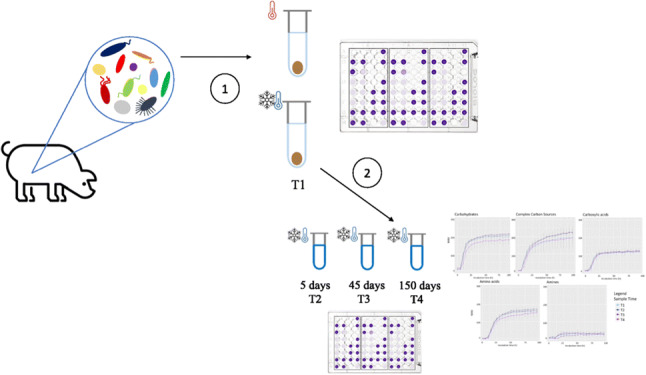

**Supplementary Information:**

The online version contains supplementary material available at 10.1007/s00253-021-11449-x.

## Introduction

Modern livestock production is based on the improvement of animal efficiency coupled with a reduction of antimicrobial use as keystones for more sustainable production of proteins of animal origin. The gut microbiota plays a key role to achieve these objectives. Indeed, the importance of the interplay between animal and gut microbiota in the regulation of the animals’ health has been widely highlighted (Putignani et al. 2014). Generally, microbial communities can contribute to the host homeostasis, affecting the host metabolism (Tremaroli and Bäckhed 2012), influencing processes including nutrient digestion, nutrient synthesis (such as vitamins and fatty acids), and gut epithelial cell turnover (Yeh et al. 2019) as well as influencing the development and activation of the immune system (Trevisi et al. 2018). In monogastric animals, microbial communities have been demonstrated to contribute to the improvement of pig feed efficiency and consequently its productive performances (Kiros et al. 2019).

Besides age and host immune competence, diet composition is one of the main factors modulating gut microbial profile and metabolism (David et al. 2014). The gut microbial community, with a huge and diverse concentration of bacteria, viruses, and fungi, contributes to the degradation of feed components during the passage of digesta along the gut intestinal tracts (GIT). Among nutrients, carbon sources and, in particular, complex carbohydrates are largely metabolized by gut microbial communities obtaining oligosaccharides, which are then fermented to short-chain fatty acids (Puertollano et al. 2014). Dietary and endogenous amino acids (AAs) can also be used by the gut microbiota (Dai et al. 2010). In the small intestine, peptides can be metabolized by bacteria resulting in the production of polyamine involved in cell metabolism, growth, and proliferation. In contrast, in the large intestine, AAs can be fermented by the resident microbiota producing detrimental metabolites such as ammonia, indole, and skatole (Bardócz et al. 1995).

Presently, several techniques have been used to investigate the animal gut microbial community composition, in particular high throughput sequencing coupled with bioinformatics analysis. Nevertheless, a proper analysis of the gut microbial community should include information regarding metabolic activity. Microbial metabolism depends on several factors, such as active population in terms of species and quantities, substrate composition, and environmental conditions; thus, meta-transcriptomics and metabolomics have been exploited to investigate the microbial community functions. An additional method to study the metabolism of entire microbial communities is represented by Biolog^TM^’s platform coupled with Ecoplates (Biolog Inc., Hayward, CA, USA), which is based on the detection and simultaneous measurement of carbon substrate utilization level from the microbial community (Stefanowicz 2006). The method has been widely used for the study of microbial metabolism of environmental sources, including soils and sediments (Gałązka et al. 2017; Lopes et al. 2016), water plants, and grains (Ge et al. 2018). Moreover, Biolog^TM^’s Ecoplates technique has been applied to evaluate indirectly the functional traits of gut microbial communities of livestock, manure composting, and wastewater (Wang et al. 2018; Gryta et al. 2014). More recently, Phenotype Microarray^TM^ was applied to study the gut and fecal microbial communities of several animal species (Grześkowiak et al. 2020; Yeh et al. 2019), including pigs (Piotrowska et al. 2014), outlining their metabolic profile in certain physiological or environmental conditions (Najdegerami et al. 2012; Pierce et al. 2016).

However, presently, there is no information regarding the effect of the conservation including freezing and length of storage of these complex matrices for their subsequent analysis through the Biolog^TM^ Ecoplates technique. This information could improve and broaden the use of this technique for the evaluation of the microbial metabolic fingerprint in pigs’ gut. In fact, in the normal day-by-day sample collection, often samples are maintained frozen before being processed. In this methodological study, the authors investigated, for the first time, the metabolic potential and the functional capacities of the microbiota of pig fecal samples using the Biolog^TM^ platform coupled with Ecoplates system with the following aims: (i) identify the optimal microbial cell concentration for metabolic function description; (ii) compare metabolic activity between fresh and frozen samples; (iii) verify that long storage time (5 months) are not significantly impacting on the correct metabolic analysis of frozen samples. Furthermore, the authors suggest the taxonomical analysis of microbiota as a method that can be coupled with the characterization of microbial community metabolic activity. Indeed, such determination allows for the direct assignment of the metabolic phenotype of the community to specific bacterial groups, which are found to be the most part in pig gut microbiota.

## Materials and methods

### Animal housing, sample collection, preparation, and studies design

Healthy post-weaning piglets (weaned at 28 days of age) were included in the experiment and housed in pens at the University of Bologna. Room temperature was kept controlled at 30 °C at the beginning and 25 °C at the end of the experiment, with a 1 °C decrease every 3 days. Infrared lamps were located above the piglets for the first 7 days post-weaning. Piglets were fed a standard commercial diet without antimicrobials and based on barley, wheat, corn, milk whey powder, and soy protein concentrate (diet composition: 17.9% crude protein and 5.0% crude fibers). Piglets had free access to feed and water throughout the experimental period; feed was ad libitum supplied in a dry feeder.

On day 35 of age, fresh feces samples were collected into a sterile collection tube of 120 mL immediately after defecation from healthy piglets. Then, to limit the environmental contamination, approximately 5g of feces was taken from the non-superficial part of the feces, homogenized, and divided into aliquots of 500 mg each. For experiment 1, two aliquots were prepared from one homogenized fecal sample: the first aliquot was stored at room temperature for 4 h (fresh sample) and the second aliquot was immediately frozen in liquid nitrogen for 5 min and then stored at −80°C (frozen sample). Both samples were analyzed after 4 h. For experiment 2, one homogenized fecal sample was divided into four aliquots that were immediately frozen in liquid nitrogen for 5 min and then stored at −80°C.

#### Experiment 1: comparison between fresh and frozen feces

Fresh and frozen samples (500 mg each) were first suspended in 50 mL sterile PBS (pH 7.2) and vortexed: these suspensions were named stock suspensions (SSUSPs). SSUSPs were shaken for 30 min at 21 °C and then allowed to rest for 15 min at 4°C, according to Ecoplates’ sample preparation protocol used by Gryta et al. (2014) and Núñez-Díaz et al. (2017). For each SSUSP, two different dilutions were prepared: 1:10 (approximately, 9 × 10^4^ bacterial cells/g) and 1:100 (approximately, 9 × 10^3^ bacterial cells/g). The bacterial cell count of SSUSP samples was performed on Luria Bertani (LB) plates, after an incubation of 24h at 39 °C. The SSUSP and the diluted samples were then analyzed using Biolog^TM^ Ecoplates. Dilution showing the highest metabolic activity was the basis for choosing the dilutions of experiment 2.

#### Experiment 2: effect of storage time

Four frozen aliquots were used for experiment 2. Bacterial metabolic activity of each different frozen aliquot was analyzed at different time points: after 1 day (T1), 5 days (T2), 45 days (T3), and 150 days (T4) of frozen storage. Based on the results obtained from experiment 1, every SSUSP was diluted to 1:2 and 1:5 (approximately 4.5 × 10^5^ bacterial cells/g and 1.8 × 10^5^ bacterial cells/g, respectively.)

### Biolog^TM^ phenotype assays

The carbon source utilization profile of pig fecal microbiota was assessed by using the fully automated Biolog Omnilog^TM^ (Biolog, Hayward, CA, USA) System through Ecoplates Phenotype MicroArray (PM). These 96-well microplates contain three replicate sets of 31 lyophilized relevant C substrates, along with a tetrazolium redox dye (Insam 1997). C substrates were grouped by chemical class (eight carbohydrates, eight carboxylic acids, four polymers, six amino acids, two amines, and three miscellaneous substrates; Table 1). Substrate utilization by the microbial community is evidenced by a color change in each plate well, as the redox dye is reduced to tetrazolium violet. Redox technology used by the PM system measures cell energy (NADH) production following substrate utilization as a universal reporter. More specifically, if the microbial cells have an active metabolism, there would be a flow of electrons to NADH, which determines the reduction of a tetrazolium dye and the consequent production of a purple color.

**Table 1:** Biochemical classification of different carbon sources

Biochemical classification	Compounds
Carbohydrates	β-Methyl-D-glucoside
	D-Xylose
	*i*-Erythritol
	D-Mannitol
	*N*-Acetyl-D-glucosamine
	D-Cellobiose
	Glucose-1-phosphate
	α-D-Lactose
	D,L-α-Glycerol phosphate
Carboxylic acid	D-Galactonic acid γ-lactone
	Pyruvic acid methyl ester
	D-Galacturonic acid
	2-Hydroxy benzoic acid
	4-Hydroxy benzoic acid
	γ-Amino butyric acid
	D-Glucosaminic acid
	Itaconic acid
	α-Keto butyric acid
	D-Malic acid
Complex carbon sources	Tween 40
	Tween 80
	α-Cyclodextrin
	Glycogen
Amino acids	L-Arginine
	L-Asparagine
	L-Phenylalanine
	L-Serine
	L-Threonine
	Glycyl-L-glutamic acid
Amines	Phenylethylamine
	Putrescine

For the study design, samples were diluted according to the experimental design previously described and 150 μl of sample supernatant was dispensed into each well of the Ecoplates (Piotrowska et al. 2014).

The Ecoplates were then incubated at 39°C in aerobic conditions in the dark. Substrate’s consumption was recorded by the automated Omnilog^TM^ System every 15 min for 96 h, to allow reaching plateau phase when the substrates are degraded. Microbial activity in each microplate well was expressed as metabolic activity value (MAV), which is the Biolog^TM^ index based on the ability to use different compounds by a bacterial community.

### Microbial community profiling

Total bacterial DNA for microbiota profile analysis was extracted from the frozen fecal samples used in experiment 2 by using FastDNA^TM^ Spin Kit for Soil (MP Biomedicals Europe, LLC). DNA quantity and quality were evaluated using NanoDrop ND-1000 spectrophotometer (NanoDrop Technologies Inc., Wilmington, DE, USA). The V3–V4 hypervariable regions of the 16S rRNA gene were then amplified with the primers Pro341F: 50-TCGTCGGCAGCGTCAGATGTGTATAAGAGACAGCCTACGGGNBGCASCAG-30 and Pro805R: 50-GTCTCGTGGGCTCGGAGATGTGTATAAGAGACAGGACTACNVGGGTATCTAATCC-30 using the Platinum^TM^ Taq DNA Polymerase High Fidelity (Thermo Fisher Scientific, Milan, Italy). Thermocycler settings: Initial denaturation at 94°C for 1 min, 25 cycles of 94°C for 30 s, 55°C for 30 s, 68°C for 45 s, and final elongation at 78°C for 7 min. The libraries were prepared using the standard protocol for MiSeq Reagent Kit v3 and sequenced on the MiSeq platform (Illumina Inc., San Diego, CA, USA).

### Statistical and bioinformatics analysis

#### Biolog^TM^ Ecoplates

Experiments were performed in duplicate, and the results were expressed as means and standard deviations. Biolog Data Analysis 1.7.51 (Biolog, Hayward, CA, USA) was used to convert data from Biolog^TM^ format to MAVs, normalizing data on the average of technical replicates and subtracting blank from the detected activity. The MAV average and standard deviation of fresh and frozen samples at different dilutions were detected for different carbon source categories (carbohydrates, complex carbon sources, carboxylic acids, amino acids, and amines) at different time points. Data after 96 h of incubation were then statistically analyzed. Data of experiment 1 were fitted using a linear model where sample type (fresh vs frozen) and dilution were included as factors. Data of experiment 2 were fitted using a linear model in which the time of storage, dilution, and their interaction were included as factors. ANOVA and Tukey’s test analysis were then carried out. The packages “car” (version 3.0.3), “lsmeans” (version 2.30.0), and “multcomp” (version 1.4.10) within the R software (R Core Team, 2019) were used to carry out the statistical analysis and “ggplot2” (version 3.3.0), “plyr” version (1.8.6), “tidyr” (version1.0.2) packages were used to create figures.

Results were considered significant at *P* ≤ 0.05 and tendencies at 0.05 ≤ *P* ≤ 0.10.

#### Next-generation sequencing analysis

Microbial sequence data were analyzed using the DADA2 pipeline (Callahan et al. 2016), and taxonomic categories were assigned by using the Silva database (release 132) as reference (Quast et al. 2012). To explore the metabolic potential of the microbial communities, a functional prediction based on the 16S rRNA gene sequences was performed using Tax4Fun (Aßhauer et al. 2015) that provides a good approximation to functional profiles obtained from metagenomic shotgun sequencing approaches. Tax4fun produces a KEGG Orthology (KO) abundance table, whereas KO identifies an ortholog of an experimentally characterized gene/protein. Subsequently, the KO enrichment analysis was carried out using Microbiome Analyst online tool (Dhariwal, et al. 2017). Enrichment analysis was performed via over-representation analysis (ORA), using the hypergeometric test to test if a particular group of KOs are represented more than expected by chance within the uploaded list of genes. *P* values were corrected for multiple comparisons using Benjamini and Hochberg’s false discovery rate (FDR). Results were considered significant at FDR < 0.05.

## Results

### Experiment 1: effect of sample storage and cell concentration on functional metabolic activity

The effect of the storage conditions (fresh sample vs frozen sample), as well as of the different bacterial cells’ concentration (9 × 10^5^ cells/g (SSUSP sample), 9 x 10^4^ cells/g (1:10 dilution), and 9 × 10^3^ cells/g (1:100 dilution)) on the functional metabolic activity (expressed as MAVs) of fecal samples was evaluated.

Supplemental Fig. S1 shows the functional metabolic diversity of fresh and frozen samples at each dilution tested for all the time points during the 96-h incubation time on each carbon source category. Results highlighted the utilization of the complex carbon sources, carboxylic acids, amino acids, and amines in both fresh and frozen sample dilutions except for dilution 1:100 in frozen condition, in which MAVs remained close to zero. Generally, lower MAVs were observed for the fresh sample diluted at 1:100 dilution compared with SSUSP and 1:10 dilution. In wells filled with carbohydrates, complex carbon sources, and carboxylic acids, the MAVs reached by the fresh sample at the dilution 1:10 at 96 h were numerically higher in respect to the MAVs of the frozen sample at the same dilutions. In wells filled with amines and amino acids, a different trend was observed: the SSUSP sample reached the highest MAVs already after 25 h of incubation (Supplemental Fig. S1).

Table 2 shows the mean of MAVs for the different carbon source classes and compounds and the effect of sample conservation (fresh vs frozen) and sample dilution after 96 h of incubation time. At the end of incubation, the results indicated that, besides the sample storage and bacterial cell concentration, the microbial community in fecal samples could not utilize or poorly utilized (< 40 MAVs) the following substrates as a carbon source: erythritol (except for fresh 1:10 sample), 2-hydroxy benzoic acid, 4-hydroxy benzoic acid, itaconic acid, and keto butyric acid. On the other hand, the sources that had higher values were pyruvic acid methyl ester, D-xylose, D-galacturonic acid, L-asparagine, *N*-acetyl-D-glucosamine, glycogen, D-cellobiose, D-xylose, and D-lactose (Table 2). The sample storage condition did not significantly affect MAVs for any of the biochemical classes and the single compounds except for Tween 80 (*P* = 0.05). In addition, the sample dilutions tended to influence the carboxylic acids and amines MAVs (*P* < 0.1). Considering the single compounds, dilutions significantly influenced the use of D-galactonic acid lactone (*P* = 0.02), pyruvic acid methyl ester (*P* = 0.04), D-galacturonic acid (*P* = 0.03), L-phenylalanine (*P* = 0.01), Tween 80 (*P* < 0.001), *N*-acetyl-D-glucosamine (*P*=0.03), glycogen (*P* = 0.03), and glucose-1-phosphate (*P* = 0.03), and tended to influence β-methyl-D-glucoside (*P* = 0.07), L-asparagine (*P* = 0.07), α-amino butyric acid (*P* = 0.06), α-keto butyric acid (*P* = 0.06), and phenylethylamine (*P* = 0.06). The samples diluted 1:10 showed higher MAVs for D-galacturonic acid, pyruvic acid methyl ester, D-galacturonic acid, *N*-acetyl-D-glucosamine, and glycogen compared with SSUSP and 1:100 samples (*P* < 0.05). The SSUSP samples had higher MAVs for L-phenylalanine and Tween 80 compared with 1:10 and 1:100 samples (*P* < 0.05). The glucose-1-phosphate was higher in both SSUSP and 1:10 samples compared with the 1:100 samples (*P* < 0.05) (Table 2).

**Table 2:** Metabolic activity values (MAVs) in different carbon sources according to sample types and sample dilution

Item	Frozen			Fresh			Conservation (frozen vs fresh)		Dilution	
	SSUSP	1:10	1:100	SSUSP	1:10	1:100	*P* value	SE	*P* value	SE
Biochemical classification										
Carbohydrates	143.00	198.08	3.69	75.60	243.28	118.93	0.62	37.60	0.24	46.10
Carboxylic acids	64.60	81.82	2.07	48.05	128.70	2.14	0.65	13.40	0.09	16.60
Amino acids	144.19	40.69	4.44	67.64	143.65	2.35	0.89	36.80	0.40	45.10
Complex carbon sources	126.18	85.76	7.65	98.10	234.65	8.11	0.54	38.80	0.27	47.50
Amines	73.06	0.00	0.19	66.75	28.53	2.78	0.51	7.39	0.06	9.05
Compounds										
β-Methyl-D-glucoside	151.01	144.16	2.35	99.33	183.88	0.00	0.87	18.70	0.07	22.90
D-Galactonic acid lactone	0.00^a^	277.53^b^	3.05^a^	85.47^a^	305.03^b^	1.15^a^	0.29	18.20	0.02	22.20
L-Arginine	145.00	8.22	8.36	0.00	0.00	0.94	0.36	32.30	0.50	38.60
Pyruvic acid methyl ester	142.91^a^	260.19^b^	5.18^a^	74.72^a^	320.79^b^	0.00^a^	0.92	26.30	0.04	32.20
D-Xylose	158.96	273.77	30.85	94.63	303.87	298.27	0.51	69.80	0.50	85.50
D-Galacturonic acid	167.21^a^	280.52^b^	4.77^a^	97.34^a^	313.27^b^	0.00^a^	0.69	21.20	0.03	26.00
L-Asparagine	179.88	235.91	6.79	107.26	312.26	1.19	0.99	30.50	0.07	37.30
Tween 40	37.41	0.00	0.00	104.62	290.80	3.50	0.30	61.60	0.52	75.40
*i*-Erythritol	0.00	0.00	0.00	0.00	141.49	0.00	0.42	33.30	0.50	40.80
2-Hydroxy benzoic acid	0.00	0.00	6.37	8.04	9.78	2.07	0.42	3.13	0.98	3.48
L-Phenylalanine	74.07^a^	0.00^b^	2.32^b^	82.45^a^	15.66^b^	0.00^b^	0.30	3.69	0.01	4.52
Tween 80	98.90^a^	39.47^b^	0.00^b^	110.95^a^	50.00^b^	21.66^b^	0.05	2.46	0.00	3.02
D-Mannitol	141.28	238.53	0.00	79.49	257.95	227.24	0.55	60.90	0.47	74.50
4-Hydroxy benzoic acid	0.00	0.00	1.30	0.00	15.51	0.00	0.47	3.82	0.54	4.68
L-Serine	171.94	0.00	4.57	102.12	268.21	6.20	0.58	72.70	0.58	89.10
α-Cyclodextrin	172.43	0.00	0.00	93.28	295.39	0.00	0.59	80.60	0.60	98.70
*N*-Acetyl-D-glucosamine	168.83^a^	311.95^b^	0.00^a^	79.71	300.00^b^	2.05^a^	0.36	20.00	0.03	24.50
α-Amino butyric acid	115.89	0.00	0.00	78.60	16.91	0.00	0.71	11.30	0.06	13.90
L-Threonine	115.46	0.00	2.39	21.09	14.68	0.32	0.56	24.00	0.39	29.40
Glycogen	175.83^a^	303.57^b^	0.00^a^	83.55^a^	302.40^b^	7.29^a^	0.46	22.50	0.03	27.60
D-Glucosaminic acid	56.99	0.00	0.00	0.00	24.04	0.00	0.69	17.00	0.68	20.80
Itaconic acid	0.00	0.00	0.00	32.26	13.90	6.58	0.15	5.40	0.50	6.62
Glycyl-L-glutamic acid	182.97	0.00	2.21	92.91	251.11	5.44	0.64	72.00	0.59	88.20
D-Cellobiose	173.89	192.31	0.00	85.93	304.62	241.37	0.45	67.70	0.58	83.00
Glucose-1-phosphate	180.16^a,b^	289.00^a^	0.00^b^	80.97^a,b^	259.76^a^	0.19^b^	0.28	20.80	0.03	25.50
α-Keto butyric acid	18.65	0.00	0.00	38.03	5.88	6.97	0.13	3.06	0.06	3.75
Phenylethyl-amine	105.72	0.00	0.37	77.05	18.58	5.56	0.92	9.94	0.06	12.20
α-D-Lactose	166.51	249.18	0.00	75.16	248.46	245.36	0.66	71.10	0.58	87.10
D,L-Glycerol phosphate	165.71	83.84	0.00	85.22	189.48	55.85	0.67	39.30	0.39	48.20
D-Malic acid	149.88	0.00	0.00	66.03	261.85	4.65	0.61	73.30	0.63	89.80
Putrescine	44.90	0.00	0.00	56.44	38.47	0.00	0.28	8.06	0.13	9.87

*SSUSP*, stock suspension, *SE*, standard error

The approximate bacterial cell concentration able to retain a detectable metabolic activity by Phenotype Microarray^TM^ technology resulted in the 9 × 10^5^ (SSUSP) and 9 × 10^4^ bacterial cells/g (1:10 dilution). Based on the obtained data, to set up experiment 2, appropriate dilutions were chosen.

### Experiment 2: preservation of the functional metabolic activity in samples with different bacterial cell concentrations and time of storage

Experiment 2 allowed to verify the impact on metabolic activity of low temperature storage at different time points (T1 = 1 day; T2 = 5 days; T3 = 45 days; T4 = 150 days) using three different bacterial concentration (SSUSP and dilutions 1:2 and 1:5).

**Fig. 1:**
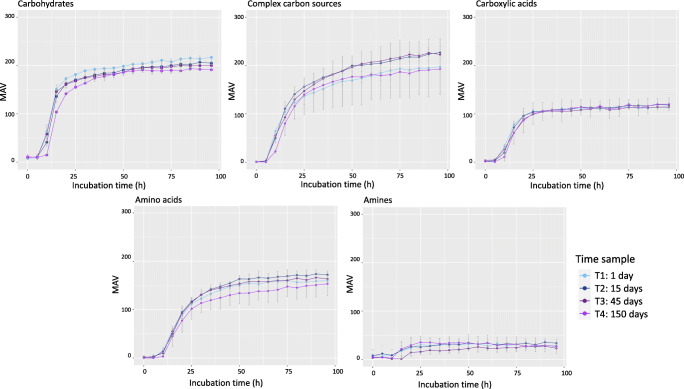
Functional metabolic diversity in the three stored samples. Metabolic activity values (MAVs) for different carbon source categories (carbohydrates, complex carbon sources, carboxylic acids, amino acids, and amines) at different time points. Dilution 1:2 of samples T1 (1 day), T2 (15 days), T3 (45 days), and T4 (150 days) is represented

**Fig. 2:**
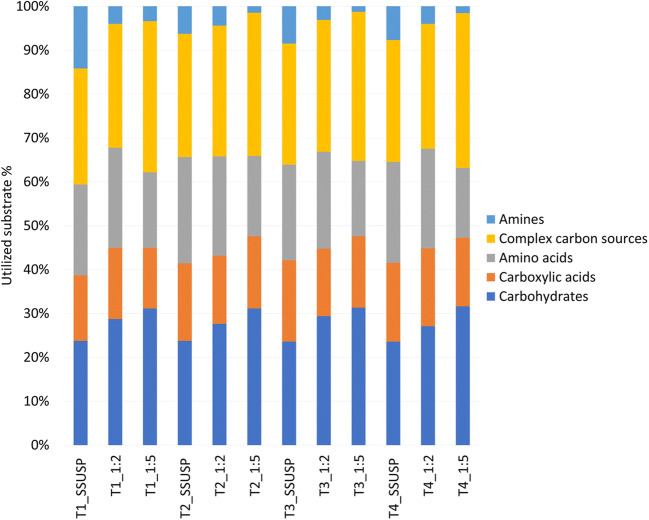
Usage of Biolog^TM^ Ecoplates substrates by the microbial community in fecal samples of the pig after 96 h of incubation

Figure 1 and Supplemental Fig. S2A and S2B show the functional metabolic diversity (represented as MAVs), during the incubation time, of 1:2 and 1:5 dilution of SSUSP sample, respectively. The storage time (T1, T2, T3, and T4) for every carbon source was evaluated. The results highlighted the utilization of carbon source in all dilutions and storage time of the samples (Fig. 1). At the end of 96 h of incubation, for both the sample dilutions and timing of storage, the MAVs of carbohydrates and complex carbon sources were the highest among the biochemical categories while those related to amines were the lowest (Fig. 2).

Table 3 reports the MAVs’ mean of the different carbon sources after 96 h of incubation, the effect of sample dilution, length of storage, and the interaction between dilution and length of storage factors.

**Table 3:** Effect of time of storage and sample dilution on the metabolic activity with the different class of sources used by piglet fecal microorganisms

Biochemical classification	SSUSP sample dilution				1:2 sample dilution				1:5 sample dilution				SE	*P* value		
	T1	T2	T3	T4	T1	T2	T3	T4	T1	T2	T3	T4		Dilution	Time	Time × dilution
Carbohydrates	144.00	156.80	114.10	86.90	200.90	210.10	217.50	183.20	216.90	204.80	199.90	191.20	10.90	0.002^a^	0.667	0.145
Carboxylic acids	90.40	116.60	89.50	66.10	113.60	118.30	114.20	120.00	96.40	108.20	103.80	94.60	8.96	0.211	0.945	0.247
Amino acids	125.30	159.50	105.30	84.80	159.70	172.10	163.70	153.30	119.90	120.20	109.50	96.40	13.10	0.120	0.728	0.400
Complex carbon sources	160.00	185.00	134.00	102.00	197.00	227.00	223.00	193.00	240.00	215.00	217.00	213.00	14.30	0.008^b^	0.280	0.130
Amines	85.95	41.53	41.02	28.35	28.07	33.57	23.00	26.98	23.66	9.65	8.04	9.47	13.30	0.012^c^	0.954	0.481

*T1*, 1 day after sampling; *T2*, 15 days after sampling; *T3*, 45 days after sampling; *T4*, 150 days after sampling, *SE*, standard error

^a^T1 = 1:2 sample dilution vs SSUSP sample, *P* = 0.0072; 1:5 sample dilution vs SSUSP sample, *P* = 0.0015; T2 = 1:2 sample dilution vs SSUSP sample, *P* = 0093; 1:5 sample dilution vs SSUSP sample, *P* = 0.0150; T3: 1:2 sample dilution vs SSUSP sample, *P* = 0.0002; 1:5 sample dilution vs SSUSP sample, *P* = 0.0005; T4: 1:2 sample dilution vs SSUSP sample, *P* = 0.0003; 1:5 sample dilution vs SSUSP sample, *P* = 0.0002

^b^T1: 1:5 sample dilution vs SSUSP sample, *P* = 0.0056; T3: 1:2 sample dilution vs SSUSP sample, *P* = 0.0042; 1:5 sample dilution vs SSUSP sample, *P* = 0.0052; T4: 1:2 sample dilution vs SSUSP sample, *P* = 0.0042; 1:5 sample dilution vs SSUSP sample, *P* = 0.0023

^c^T1: 1:2 sample dilution vs SSUSP sample, *P* = 0.062; 1:5 sample dilution vs SSUSP sample, *P* = 0.062

No significant effect for the storage duration was observed even though a decrease of the MAVs, especially those of amine utilization, was found in all the time points studied for SSUSP and 1:5 dilutions of all samples. Instead, sample dilution significantly affected the MAVs of carbohydrates (*P* = 0.002), complex carbon sources (*P* = 0.008), and amines (*P* = 0.0012). For carbohydrates, both dilutions 1:2 and 1:5 showed higher MAVs than the SSUSP sample during all the duration of storage (*P* < 0.05). For complex carbon sources, the 1:2 dilution reached higher MAVs than the SSUSP sample at T3 (*P* = 0.004) and T4 (*P* = 0.004). Moreover, the 1:5 dilution showed higher MAVs than SSUSP sample at T1 (*P* = 0.0056), T3 (*P* = 0.0052), and T4 (*P* value = 0.0023). For the amines, the SSUSP sample tended to have higher MAVs than dilutions 1:2 and 1:5 at T1 (*P* = 0.062).

The MAVs of each single compound for the samples at the different storage times and dilutions and for three different time points of incubation were shown in three heatmaps (Supplemental Fig. S3). The 20-, 50-, and 96 h incubation times were chosen to capture the initial, the increasing, and the plateau MAV levels. As depicted in Supplemental Fig. S3, the fecal microbiota metabolized a number of the compounds at different rates, according to the dilution. The time of sample storage did not significantly affect the fecal sample’s activities. The SSUSP samples showed a lower metabolic activity at all the time points of Ecoplates incubation compared with 1:2 and 1:5 dilutions. After 96 h of incubation, the results indicated that, besides the timing of storage, in the samples diluted to 1:2 and 1:5, the fecal microbial community could not use the following compounds as the carbon source: 2-hydroxy benzoic acid, α-keto butyric acid, and the 4-hydroxy benzoic acid. The sources which showed higher MAVs were β-methyl-D-glucoside, L-threonine, Tween 40, D-mannitol, X D-lactose, D,L-glycerol phosphate, L-serine, glycyl-L-glutamic acid, Tween 80, X-cyclodextrin, D-xylose, glucose-1-phosphate, glycogen, D-galactonic acid lactone, D-malic acid, pyruvic acid methyl ester, D-galacturonic acid, *N*-acetyl D-glucosamine, D-cellobiose, and L-asparagine.

Table 4 reports the effect of storage time, sample dilution, and their interaction on the different sources after 96 h of incubation. Overall, the time of storage did not significantly affect the metabolic activity of the fecal microbial profile. The interaction between time of storage and sample dilution was significant or tended to be significant for *i*-erythritol (*P* = 0.025), 4-hydroxy benzoic acid (*P* = 0.08), and itaconic acid (*P* = 0.025).

**Table 4:** Effect of time of storage and dilution of the samples on the different compounds used by bacteria community in pigs’ fecal samples

Biochemical classification	SSUSP sample dilution				1:2 sample dilution				1:5 sample dilution				SE	*P* value		
	T1	T2	T3	T4	T1	T2	T3	T4	T1	T2	T3	T4		Dilution	Time	Time × dilution
Negative control	0.03	0.02	0.01	0.03	0.01	0.03	0.00	0.02	0.02	0.01	0.05	0.00	0.01	0.669	0.387	0.090
β-Methyl-D-glucoside	133.00	152.50	98.30	85.00	181.10	170.30	149.80	139.70	149.30	125.30	141.40	158.50	24.00	0.388	0.620	0.568
D-Galactonic acid γ-lactone	162.00	185.00	136.00	100.00	239.00	252.00	229.00	216.00	244.00	260.00	257.00	230.00	11.70	0.001	0.220	0.254
L-Arginine	9.10	100.90	0.00	0.00	17.90	14.80	20.60	15.80	27.20	21.90	21.30	28.20	31.00	0.919	0.999	0.527
Pyruvic acid methyl ester	159.00	181.00	132.00	104.00	240.00	254.00	238.00	216.00	260.00	274.00	261.00	241.00	12.50	<0.0005	0.247	0.550
D-Xylose	162.00	174.00	126.00	101.00	234.00	249.00	234.00	222.00	254.00	261.00	257.00	252.00	10.80	<0.0005	0.408	0.109
D-Galacturonic acid	164.00	183.00	133.00	102.00	244.00	259.00	237.00	221.00	264.00	270.00	257.00	248.00	13.60	0.001	0.294	0.462
L-Asparagine	170.00	193.00	137.00	113.00	245.00	254.00	233.00	219.00	243.00	237.00	222.00	228.00	15.20	0.007	0.419	0.460
Tween 40	159.60	186.00	133.50	103.90	200.20	166.00	214.80	158.00	183.10	91.70	128.70	116.50	18.60	0.337	0.154	0.069
*i*-Erythritol	50.07	0.00	21.10	13.83	12.54	18.38	192.69	54.57	6.97	0.55	0.00	0.00	18.90	0.256	<0.0001	0.002
2-Hydroxy benzoic acid	0.00	0.00	0.00	0.00	0.00	0.00	0.00	0.00	6.97	0.00	0.00	0.00	2.01	0.049	1.000	0.472
L-Phenylalanine	114.93	112.10	105.01	84.57	41.89	48.64	77.03	88.13	8.87	0.00	0.00	0.00	16.50	0.003	0.204	0.361
Tween 80	157.90	186.70	133.40	98.50	232.30	229.00	205.10	203.20	266.40	236.50	222.80	227.70	12.90	<0.0005	0.287	0.165
D-Mannitol	149.50	173.40	122.40	84.60	204.50	239.70	217.30	186.60	230.20	219.00	217.20	210.20	15.90	0.012	0.174	0.303
4-Hydroxy benzoic acid	30.46	21.51	22.51	17.14	1.20	0.00	6.91	43.79	3.31	0.00	0.00	0.00	8.47	0.059	0.011	0.084
L-Serine	164.00	186.00	136.00	107.00	227.00	248.00	228.00	215.00	250.00	255.00	236.00	230.00	11.90	0.001	0.316	0.339
α-Cyclodextrin	162.00	185.00	134.00	101.00	233.00	254.00	230.00	204.00	240.00	247.00	249.00	245.00	13.50	0.003	0.136	0.164
*N*-Acetyl-D-glucosamine	159.30	178.50	126.00	97.80	244.10	255.20	237.90	221.80	266.10	271.80	260.50	255.20	11.50	<0.0001	0.277	0.205
γ-Amino butyric acid	132.94	196.25	121.07	102.36	77.69	90.59	101.13	111.32	9.06	0.00	0.69	4.75	14.90	<0.0005	0.454	0.037
L-Threonine	129.10	174.25	123.59	94.82	197.48	220.58	196.29	171.75	4.95	5.76	7.88	0.00	13.40	<0.0001	0.144	0.312
Glycogen	162.00	184.00	134.00	106.00	238.00	258.00	242.00	206.00	271.00	284.00	267.00	264.00	11.90	<0.0005	0.056	0.290
D-Glucosaminic acid	55.77	63.55	68.51	43.88	75.64	75.50	155.63	139.46	9.12	0.00	6.14	2.67	23.20	0.160	0.062	0.381
Itaconic acid	41.55	70.73	14.00	28.54	19.75	8.62	8.58	45.29	1.95	8.14	0.00	0.86	9.04	0.031	0.047	0.025
Glycyl-L-glutamic acid	165.20	191.00	129.90	109.50	229.20	246.20	227.70	210.00	185.80	201.30	169.70	91.40	19.00	0.093	0.623	0.421
D-Cellobiose	162.30	185.80	134.90	96.50	244.40	253.00	242.00	214.80	249.20	233.90	243.10	212.20	15.60	0.004	0.384	0.432
Glucose-1-phosphate	155.10	184.30	130.00	94.90	235.40	255.30	234.40	215.20	254.90	268.80	259.10	251.90	13.00	<0.0005	0.248	0.233
α-Keto butyric acid	10.92	75.33	95.50	61.07	0.00	0.00	6.55	0.72	3.92	0.00	0.00	0.00	7.69	0.914	0.610	0.002
Phenylethyl-amine	78.07	77.18	72.70	56.69	34.27	53.25	41.26	53.97	9.78	2.90	0.00	0.00	10.40	0.002	0.497	0.578
α-D-Lactose	162.00	190.00	140.00	110.00	226.00	228.00	209.00	192.00	236.00	259.00	235.00	226.00	13.70	0.006	0.259	0.584
D,L-α-Glycerol phosphate	162.80	172.00	128.20	98.90	226.10	221.40	193.10	202.40	189.90	202.80	185.70	154.50	18.20	0.089	0.553	0.752
D-Malic acid	148.00	190.00	128.00	101.00	239.00	242.00	215.00	207.00	256.00	269.00	256.00	219.00	15.00	0.001	0.300	0.565
Putrescine	93.83	5.87	9.34	0.00	21.87	13.89	4.73	0.00	37.54	16.40	16.08	18.94	25.00	0.147	0.926	0.657

*T1*, 1 day after sampling; *T2*, 15 days after sampling; *T3*, 45 days after sampling; *T4*, 150 days after sampling

Furthermore, the dilution of the samples significantly affected the metabolic activity profile of the piglet fecal microbial community; the MAVs of the 1:2 and 1:5 sample dilutions were higher than the MAVs of the SSUSP sample for the following sources: L-threonine, *N*-acetyl-D-glucosamine, glycogen, D-xylose, Tween 80, pyruvic acid methyl ester, amino butyric acid, glucose-1-phosphate, D-galactonic acid lactone, D-galacturonic acid, D-malic acid, L-serine, phenylethylamine, L-phenylalanine, cyclodextrin, D-cellobiose, D-lactose, L-asparagine, D-mannitol, itaconic acid, and 2-hydroxy benzoic acid (*P* < 0.05). β-methyl-D-glucoside, L-arginine, Tween 40, *i*-erythritol, D-glucosaminic acid, α-keto butyric acid, and putrescine were not affected by the dilution of the samples.

### Microbial community profile and functional analysis

**Fig. 3:**
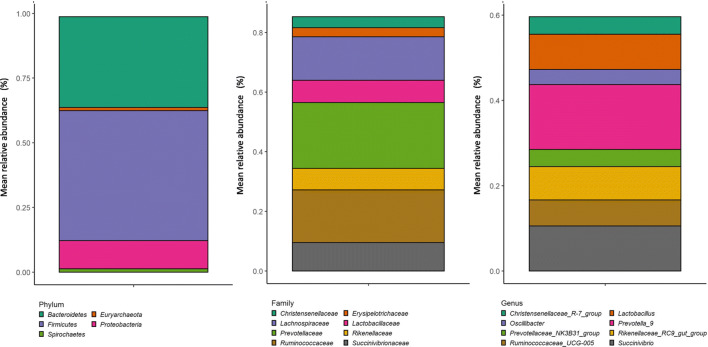
Microbial profile of pig fecal sample of experiment 2 and relative abundance at phylum (**A**), family (**B**) and genus (**C**) levels

The microbial community of the sample at T1 (frozen sample) was taxonomically analyzed. A total of 43,129 reads were attributed to a total of 3524 amplicon sequence variants (ASVs). The taxonomic assignment allowed obtaining 11 phyla, 37 families, and 73 genera. At the phylum level, Firmicutes (50%), Bacteroidetes (41%), and Proteobacteria (4%) were the most abundant; at the family level, the sample was dominated by Prevotellaceae (27%), Ruminococcaceae (17%), Lachnospiraceae (11%), and Lactobacillaceae (11%); at the genus level, *Prevotella*_9 (19%) and *Lactobacillus* (11%) were the most abundant, followed by *Rikenellaceae*_RC9_gut_group (9%), *Ruminococcaceae*_UCG_005 (6%), and *Prevotellaceae*_NK3B31_group (4%) (Fig. 3).

To investigate the microbial functional diversity, the sequences obtained from 16S rRNA sequencing were annotated to the KEGG database using Tax4fun. The KO table produced was used to perform the enrichment analysis, to identify pathways that are represented more than expected by chance. The results of the enrichment analysis are reported in Supplemental Table S1. The most enriched pathway resulted in the biosynthesis of amino acids, carbon metabolism, methane metabolism, porphyrin and chlorophyll metabolism, carbon fixation pathways, pyruvate, propanoate, glycine, serine and threonine metabolism, peptidoglycan biosynthesis, and glycolysis/gluconeogenesis (*P* FDR < 0.01).

## Discussion

Compared to meta-transcriptomics, metabolomics, and other tools generally applied to investigate the microbial community functions, the Phenotype Microarray^TM^ is a relatively fast and cost-effective method to examine microbial metabolic functions and phenotypes. In particular, the Ecoplates technique enables the study of whole microbial communities and investigating their carbon substrate utilization, outlining a community metabolic fingerprinting.

The present study evaluated the inoculum density and the storage conditions needed to preserve a detectable metabolic activity of the microbial community present in piglet fecal specimens.

A significant effect of the inoculum density on the consumption of the carbon substrates was observed. Indeed, considering the results of experiment 1, only the dilutions of the original fecal sample, which contained about 9 × 10^5^ bacterial cells/g and the dilution containing 9 × 10^4^ bacterial cells/g showed a detectable functional activity. Furthermore, differently diluted samples showed differences in the metabolic performances for carboxylic acids (higher in 9 × 10^4^ bacterial cells/g dilution in respect to SSUSP sample) and amines consumption (lower in 9 × 10^4^ bacterial cells/g dilution in respect to SSUSP sample). These results suggested that not only the composition of the microbial population, as already described by Szabó et al. (2007), but also the total cell concentration of the microbial community and the growth condition in the presence of O_2_ can affect its general functions and metabolism (for example, its ability to degrade organic matter). Indeed, it is possible that a large number of cells (bacterial, but not only) can generate a premature saturation of the Ecoplates system, rapidly consuming the substrates in the wells. This may generate a pH variation counterproductive to microbial growth and the Biolog^TM^ staining measurement (Biolog, personal communication). Therefore, it is usual that undiluted inoculum shows the worst growth performances than diluted ones. Microbiologically, dilution cultures have proven advantageous in the study of oligotrophic bacteria, since inoculum dilution maintains numerically abundant organisms while eliminating fast-growing competitors that typically prevail in enrichment cultures (De Fede et al. 2001).

A potential more uniform response for all the categories of compounds was found with an inoculum density of 9 × 10^5^ (SSUSP) and 9 × 10^4^ (1:10) cells/g; however, the dilution 1:10 showed a lower MAV trend for amines and amino acids compared to SSUSP samples that in the other side had a lower trend for carbohydrates; therefore, two intermediate dilutions 1:5 (1.8 × 10^5^ cells/g) and 1:2 (4.5 × 10^5^ cells/g) were utilized in experiment 2 to optimize the metabolic activity detection.

Recently, the study of Metzler-Zebeli et al. (2016) showed that, in pig feces, the freezing process can influence the DNA yield and bacterial abundances, while the snap freezing and storage temperature had only little effect on the bacterial abundance. Therefore, this study evaluated the type of storage and the preservation of the bacterial community activity for 5-month storage at −80 °C.

Considering the optimal dilutions of the samples, the results showed no difference in the metabolic activities of the pig fecal microbial community when the same inoculum density was compared in both fresh and frozen samples. Therefore, the immediate freezing and storage at −80°C of pig fecal samples did not affect the microbial metabolic potential and substrates utilization after 96 h of incubation. Results also suggested that sample freezing, when performed immediately after the sample collection, can ensure a robust and error-minimizing analysis of the microbial metabolism. Therefore, there is no need to process a sample immediately after collection, as reported in several studies (Piotrowska et al. 2014; Yeh et al. 2019), resulting in higher analysis flexibility and a broader application of this technique.

Furthermore, the results obtained in experiment 2 confirmed that the sample storage at −80°C for 150 days did not affect the substrate consumption by the microbial community. The utilization rate of carbon sources showed a similar trend during the incubation time at all tested microbial concentrations, and it was not influenced by the time of storage of the samples. Also, when considering the single compound utilization after 96 h of incubation, overall, the time of storage had no statistically significant effect.

Overall, in agreement with Grześkowiak et al. (2020) and Kong et al. (2013), carbohydrates were the most consumed substrate category, while the least used carbon sources were the ammines, that vary depending on the microbial community tested (Grześkowiak et al. 2020; Kong et al. 2013). The utilization of carbohydrate sources by the pig fecal microbial community was confirmed by the enrichment analysis on the list of KEGG genes predicted from the taxonomic composition of the microbial community of our sample. Indeed, our results evidenced that the major enriched pathways were related to carbohydrate. According to our Biolog^TM^ results, the most consumed carbohydrates, on which the MAVs reached after 96 h of incubation remained almost unaltered in all dilution tested, were glucose-1-phosphate, α-D-lactose, D-xylose, D-cellobiose, and *N*-acetyl-D-glucosamine. In accordance with the observation of Grześkowiak et al. (2020), our data confirmed that the primary substrate for the pig gut microbiota is glucose in its different forms. Furthermore, the always elevated consumption of lactose can be due to the high abundance of *Lactobacillus* in the fecal microbial community of our sample. This genus, which is typical of the gut microbiota of young pigs, tends to increase according to the quantity of dietary lactose that is high in the diet of young piglets (Daly et al. 2014). Accordingly, *Lactobacillus* abundance undergoes a strong reduction in growing and adult pigs (Han et al. 2018). The taxonomic composition of the microbial community observed in the present study agrees with the common fecal bacterial profile of the weaned pigs (Chen et al., 2017; Motta et al., 2019). Interestingly, the high consumption of D-cellobiose should allow hypothesizing unaltered metabolic activity of cellulolytic bacteria (Gupta et al. 2012), such as those belonging to the family of Ruminococcaceae, classically associated with ruminants’ and non-ruminants’ gut, such as horses and pigs, largely present in our samples. Another highly abundant genus in our fecal samples was *Prevotella*. *Prevotella* abundancy in the gut of post-weaned pigs is generally connected to the intake of starch and fiber-rich diet which increases in the post-weaning period (Ivarsson et al. 2014). However, its abundance is positively correlated with the content of xylose of the pigs’ diet (Ivarsson et al. 2014) and it is known that some species of *Prevotella* can utilize monosaccharides including glucose (Takahashi and Yamada 2000).

Apart from carbohydrates, the modern diets for piglets have a concentration of highly digestible crude protein of around 17% and include free amino acids to satisfy piglet nutrient requirements. Human and animal gut microbes may extensively utilize amino acids for the synthesis of proteins in a species-dependent manner. In our study, L-asparagine and L-serine were the most used amino acids. Previous studies related to the metabolism of the amino acids in microbes in the pig intestinal content demonstrated that asparagine, together with aspartate, is one of the most rapidly fermented amino acids by ileal and jejunal mixed bacteria (Dai et al. 2010). This can explain the high consumption rate of asparagine by the fecal microbes of our samples. The results of the prediction of the pig feces microbiome functions obtained from 16S rRNA sequencing revealed that the pathway of serine and threonine metabolism was one of the most represented pathways identified by Tax4fun, which confirmed recent data on the functions of the pigs’ cecum and ileum microbiome (Umu et al. 2020). In supporting this, it is known that in the large intestine of pigs a large variety of bacteria can use mucus and endogenous sources that are rich in threonine and serine (Le Floc’h et al. 2018). That information can explain the always high utilization of L-serine in all the samples and dilutions observed in the present study.

Our results showed a relatively low consumption of amines as carbon sources, which (i) further decreased at lower bacterial cell concentration (1:100 dilution) and (ii) numerically decreased with sample storage (from T1 to T4 in dilution 1:5). As for amines, the Ecoplates includes phenylethylamine and putrescine. Phenylethylamine is the decarboxylation product of phenylalanine, and it is catalyzed by the enzyme tyrosine decarboxylase (Kong et al. 2013) as a result of microbial fermentation. Recently, it has been investigated as a potential antimicrobial compound in cross-contamination of food products, influencing bacterial growth and biofilm formation through the interference with bacterial signal transduction pathways (Lynnes et al. 2014). Therefore, we can hypothesize that a similar inhibitory effect of phenylethylamine was exerted on piglets’ fecal microbiota, in particular at the lower bacterial cell concentration. Putrescine is one of the main polyamines formed in the intestinal tract from bacteria by decarboxylation of lysine and ornithine. The pathways for bacterial degradation of putrescine are related to those of γ-aminobutyric acid (GABA), arginine, and ornithine. The pathways for putrescine degradation are transaminase pathways and the glutamylation pathways (Kurihara et al. 2008), which can degrade putrescine as a sole carbon source at ≤30° C, but not at higher temperatures, as confirmed by the results of our experiments where the incubation temperature was set at 39° C to mimic the pig internal body temperature.

As previously reported, the less consumed carbon sources vary depending on the microbial community. In our study, low utilization of amines (phenylethylamine and putrescine) by the piglet fecal microbial community was observed. The main pathways of polyamine catabolism are the oxidative deamination by Cu^2+^ diamine oxidase enzyme (DAO, EC 1.4.3.22) and by the monoamine oxidase enzyme (*N*-acetylputrescine oxidase, MAO, EC 1.4.3.4). Only a few strains of lactic acid bacteria (LAB) have been proven to degrade amines (Callejón et al. 2014) and in particularly stringent pH conditions. Thus, this may explain the results obtained in the metabolic profile analysis of our samples. Indeed, we tested fecal samples of 35-day-old pigs where the microbiota profile highlighted a high relative abundance of genera belonging to the lactic acid–producing bacteria.

The present study is the first pilot methodological study aimed to characterize the phenotype of pig fecal samples’ microbial community. The results were comparable with the metagenomics data. Nevertheless, more additional aspects need to be considered. Firstly, in addition to bacteria, the microbial community of pig fecal samples is characterized by other microorganisms, such as yeasts and fungi (Ramayo-Caldas et al., 2020). This may have led to discrepancies between the experimental results of functional metabolism detected by the Ecoplates and the prediction analysis on 16S rRNA performed on the bacterial microbiota. Secondly, the aerobic incubation of sample cultures during Ecoplates experiments may have excluded the strict anaerobic microorganisms, which generally are represented in high percentage in pigs’ fecal samples and whose metabolism plays a fundamental role among gut microbes (Crespo-Piazuelo et al., 2018). Thus, further Ecoplates^TM^ experiments on fecal samples under anaerobic conditions must integrate these results and are desirable to deeper investigate the metabolic activity of microbial community simulating intestinal conditions of pigs.

In conclusion, the results obtained in this study evidenced that the functional metabolic activity of the aerotolerant microbes in pig’s fecal samples can be preserved without statistically significant variation until 150 days after a snap freezing and storage at −80°C. Therefore, the sample’s microbial community remained representative within 5 months from the sampling. Furthermore, the bacterial cell concentration, if between 4.5 × 10^5^ and 1.8 × 10^5^ bacterial cells/g, does not influence the microbial community metabolism in different carbon sources tested. These results suggested high flexibility of the analysis and broader application of this technique that can represent a rapid and representative method to explore the metabolic capabilities of the microbial community in animal samples which, coupled with the investigation of species distribution in the samples, could contribute to explain the health status and the physiology of the animals. Furthermore, the utilization of prediction methods for the functional gene family’s abundance present in the fecal microbial communities through the taxonomical characterization of bacterial community is of importance for the prediction and the comparison of the functional potential of fecal samples belonging to different individuals. The application of Biolog Ecoplates coupled with sequencing prediction method may contribute to deeply understand the interplay between gut microbiota and host, helping in moving from a sterile description of the gut microbiota to a more accurate exploitation of its potential in livestock animals.

Nevertheless, the combination of the PM technique with 16S rRNA sequencing data can present some limitations (neither can represent the totality of the metabolic capabilities of feces microbial community), it is necessary to consider that no condition (dilutions, states of conservation, temperatures, oxygenation conditions) is suitable for all microbes present in the samples. Therefore, the connection reported in this study can still represent the first attempt for a better description of gut microbiota, contributing to give a further perspective regarding the key role of gut microbiota in pigs.

In the future, for a complete assessment of metabolic activity, it will be necessary to perform Phenotype Microarray^TM^ with Ecoplates setting up both aerobic and anaerobiobic condition, coupled with a targeted and untargeted metagenomics sequencing for the taxonomical and functional characterization of such entire fecal microbial community.

## Supplementary Information

ESM 1 (PDF 962 kb)

## Data Availability

All data generated or analyzed during this study are included in this published article (and its supplementary materials file).

## Code availability

The datasets on microbiota data supporting the conclusions of this article are available in the PiGutNet area of the European Nucleotide Database (ENA) repository with the number PRJEB40788.
