# Correction to: Assessment of Biolog Ecoplate™ method for functional metabolic diversity of aerotolerant pig fecal microbiota

**DOI:** 10.1007/s00253-022-11991-2

**Published:** 2022-06-10

**Authors:** A. Checcucci, D. Luise, M. Modesto, F. Correa, P. Bosi, P. Mattarelli, Paolo Trevisi

**Affiliations:** Department of Agricultural and Food Sciences, University of Bologna, 40127 Bologna, Italy

**Correction to: Applied Microbiology and Biotechnology (2021) 105:6033–6045**

**https://doi.org/10.1007/s00253-021-11449-x**

The original version of this article contains a mistake in Fig. 1.

Below is the correct Fig. 1 image.

**Fig. 1:**
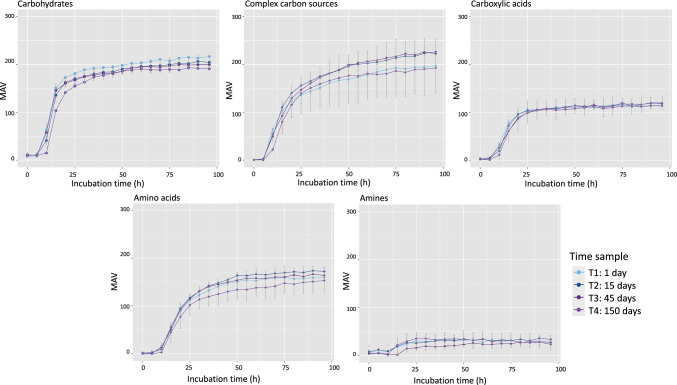
Functional metabolic diversity in the three stored samples. Metabolic activity values (MAVs) for different carbon source categories (carbohydrates, complex carbon sources, carboxylic acids, amino acids, and amines) at different time points. Dilution 1:2 of samples T1 (1 day), T2 (15 days), T3 (45 days), and T4 (150 days) is represented

This is being corrected in this publication.

