# Supplementary material for: Assessment of Biolog EcoplateTM method for functional metabolic diversity of aerotolerant pig fecal microbiota
Source: Appl Microbiol Biotechnol. 2021 Jul 23;105(14-15):6033–45. doi: 10.1007/s00253-021-11449-x (PMC8390420; doi:10.1007/s00253-021-11449-x)
Supplement: Supplementary file 1 — (PDF 962 kb) [file 253_2021_11449_MOESM1_ESM.pdf]

Supplementary materials

Journal: Applied Microbiology and Biotechnology

Assessment of Biolog Ecoplate^{TM} method for functional metabolic diversity of aerotolerant pig fecal microbiota

A. Checcucci [a1], D. Luise [a1], M. Modesto [a], F. Correa [a], P. Bosi [a], P. Mattarelli [a], P. Trevisi

[a] *Department of Agricultural and Food Sciences, University of Bologna, 40127 Bologna, Italy*

[1]*equal contribution*

Corresponding author: Paolo Trevisi. E-mail: paolo.trevisi@unibo.it.

Supplementary Table S1. Results of the pathway enrichment analysis based on bacterial 16S rRNA gene classification performed on the fecal samples of Experiment 2.

Pathway	Total[1]	Expected[2]	Hits[3]	*P*-value	FDR[4]
Biosynthesis of amino acids	222	118	169	0.00	0.00
Carbon metabolism	249	133	179	0.00	0.00
Methane metabolism	105	56	86	0.00	0.00
Porphyrin and chlorophyll metabolism	69	36.8	60	0.00	0.00
Carbon fixation pathways in prokaryotes	60	32	51	0.00	0.00
Pyruvate metabolism	74	39.5	58	0.00	0.00
Propanoate metabolism	55	29.3	45	0.00	0.00
Glycine, serine and threonine metabolism	78	41.6	58	0.00	0.00
Peptidoglycan biosynthesis	13	6.94	13	0.00	0.00
Glycolysis / Gluconeogenesis	80	42.7	58	0.00	0.00
Starch and sucrose metabolism	65	34.7	48	0.00	0.01
Glyoxylate and dicarboxylate metabolism	51	27.2	39	0.00	0.01
Fructose and mannose metabolism	44	23.5	34	0.00	0.01
Pentose phosphate pathway	63	33.6	45	0.00	0.02
Lipopolysaccharide biosynthesis	17	9.07	15	0.00	0.03
Arginine and proline metabolism	115	61.4	76	0.00	0.03
Thiamine metabolism	23	12.3	19	0.00	0.03
Terpenoid backbone biosynthesis	23	12.3	19	0.00	0.03
Pantothenate and CoA biosynthesis	29	15.5	23	0.00	0.03
Benzoate degradation	59	31.5	42	0.00	0.03
Streptomycin biosynthesis	12	6.4	11	0.01	0.04
Valine, leucine and isoleucine biosynthesis	15	8	13	0.01	0.05
Citrate cycle (TCA cycle)	53	28.3	37	0.01	0.06
Fluorobenzoate degradation	11	5.87	10	0.01	0.06
Folate biosynthesis	29	15.5	22	0.01	0.06
Amino sugar and nucleotide sugar metabolism	64	34.1	43	0.02	0.09
Lysine biosynthesis	41	21.9	29	0.02	0.09
Histidine metabolism	35	18.7	25	0.02	0.12
D-Glutamine and D-glutamate metabolism	6	3.2	6	0.02	0.12
Phenylalanine, tyrosine and tryptophan biosynthesis	64	34.1	42	0.03	0.14
Steroid degradation	9	4.8	8	0.03	0.14
Selenocompound metabolism	15	8	12	0.03	0.14
Cysteine and methionine metabolism	71	37.9	46	0.03	0.14
Butanoate metabolism	61	32.5	40	0.03	0.15
Chloroalkane and chloroalkene degradation	23	12.3	17	0.04	0.15
Xylene degradation	20	10.7	15	0.04	0.17
Synthesis and degradation of ketone bodies	5	2.67	5	0.04	0.17

[1] Number of KO in the Pathway,[2] Number of KO expected to map to the Pathway, [3]Number of KO mapped to the pathway, [4] Benjamini and Hochberg's False-Discovery Rate (FDR).

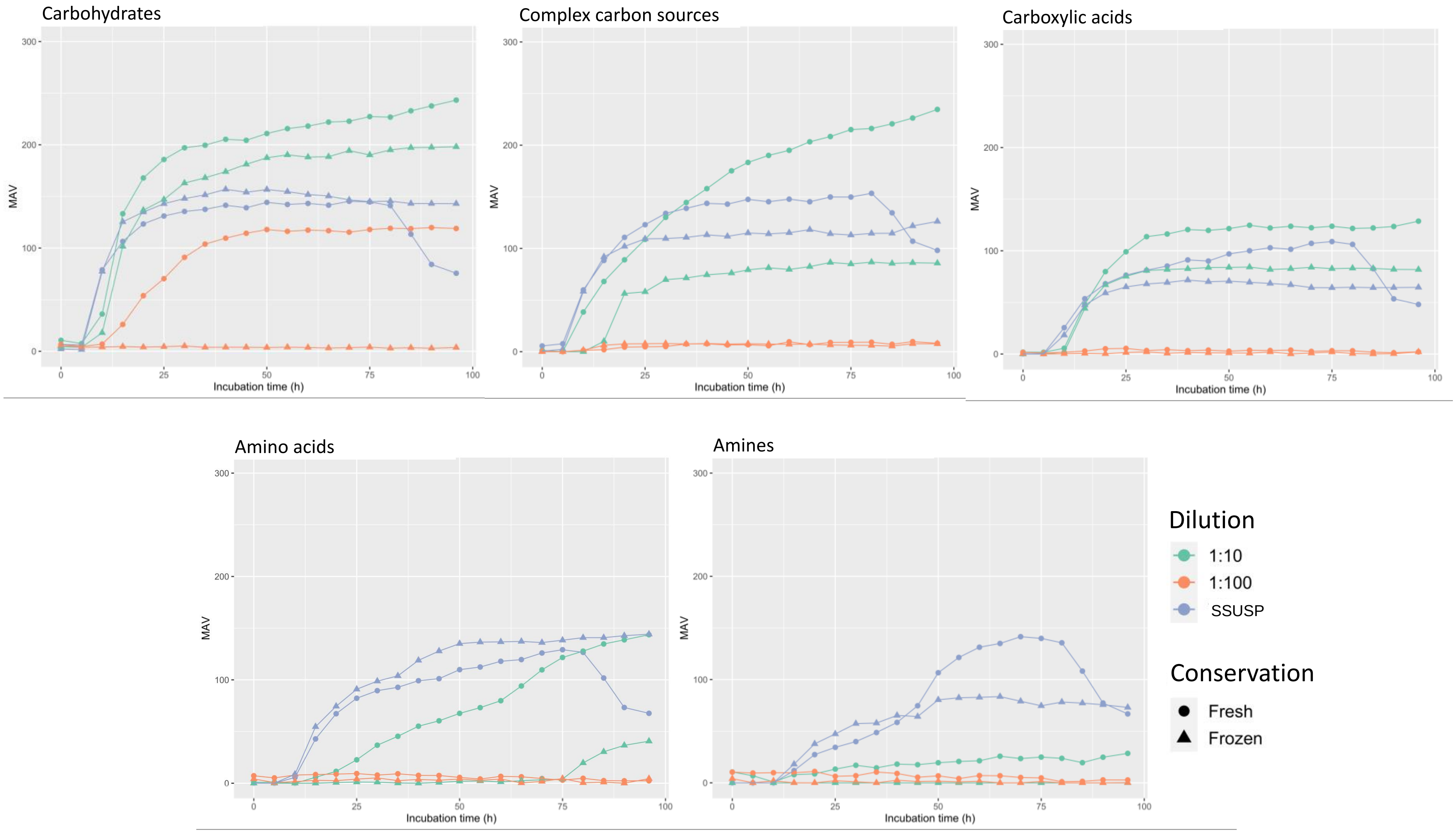

Carbohydrates
Complex carbon sources
Carboxylic acids
Amino acids
Amines
MAV
Incubation time (h)
0
25
50
75
100
200
300
Dilution
1:10
1:100
SSUSP
Conservation
Fresh
Frozen

Supplementary Fig. S1. Functional metabolic diversity in Fresh and Frozen samples in the three different dilutions tested. Metabolic Activity Values (MAV) for the different carbon sources categories (carbohydrates, complex carbon sources, carboxylic acids, amino acids and amines) at different timepoint. As indicated in the legend, dilutions are evidenced by colour and samples are represented by different timepoint shape.

A)

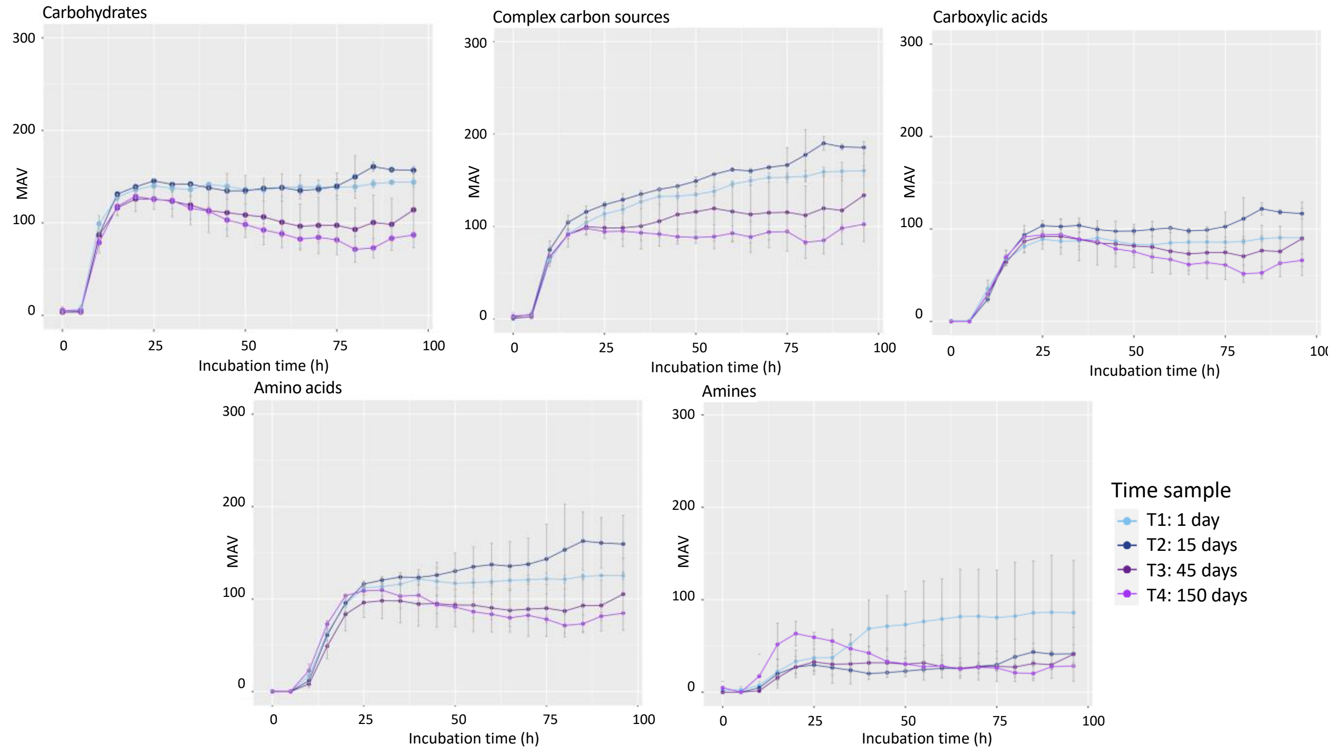

B)

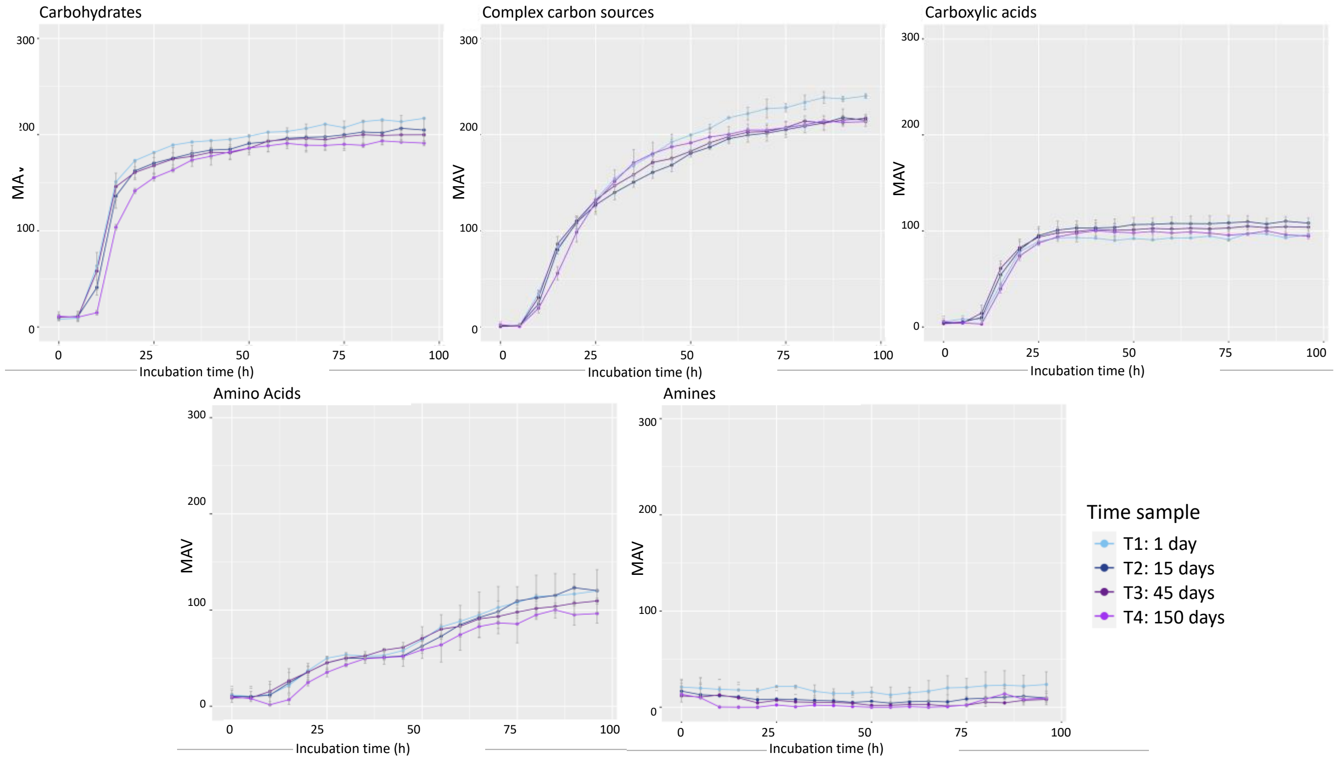

Supplementary Fig. S2. Metabolic Activity Value (MAV) for the different carbon sources categories (carbohydrates, complex carbon sources, carboxylic acids, amino acids and amines) at different timepoint. A) Undiluted samples T1, T2, T3 and T4 is represented. B) Samples diluted 1:5 at T1, T2, T3 and T4 is represented.

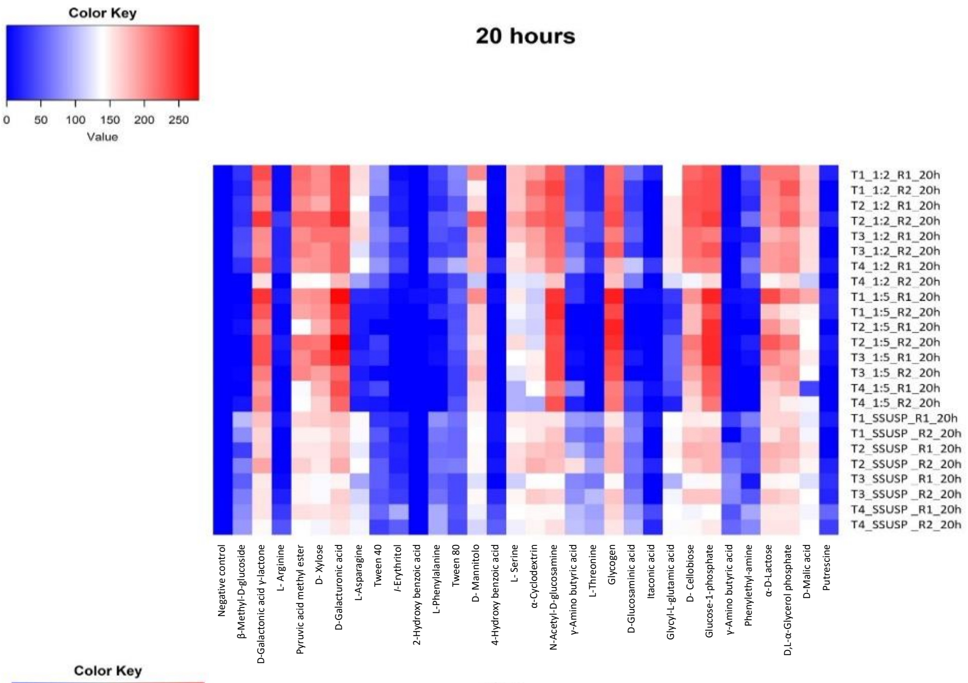
Color Key
0 50 100 150 200 250
Value
20 hours
T1_1:2_R1_20h
T1_1:2_R2_20h
T2_1:2_R1_20h
T2_1:2_R2_20h
T3_1:2_R1_20h
T3_1:2_R2_20h
T4_1:2_R1_20h
T4_1:2_R2_20h
T1_1:5_R1_20h
T1_1:5_R2_20h
T2_1:5_R1_20h
T2_1:5_R2_20h
T3_1:5_R1_20h
T3_1:5_R2_20h
T4_1:5_R1_20h
T4_1:5_R2_20h
T1_SSUSP_R1_20h
T1_SSUSP _R2_20h
T2_SSUSP _R1_20h
T2_SSUSP _R2_20h
T3_SSUSP _R1_20h
T3_SSUSP _R2_20h
T4_SSUSP _R1_20h
T4_SSUSP _R2_20h
Negative control
β-Methyl-D-glucoside
D-Galactonic acid γ-lactone
L- Arginine
Pyruvic acid methyl ester
D- Xylose
D-Galacturonic acid
L-Asparagine
Tween 40
i-Erythritol
2-Hydroxy benzoic acid
L-Phenylalanine
Tween 80
D- Mannitolo
4-Hydroxy benzoic acid
L- Serine
α-Cyclodextrin
N-Acetyl-D-glucosamine
γ-Amino butyric acid
L-Threonine
Glycogen
D-Glucosaminic acid
Itaconic acid
Glycyl-L-glutamic acid
D- Cellobiose
Glucose-1-phosphate
γ-Amino butyric acid
Phenylethyl-amine
α-D-Lactose
D,L-α-Glycerol phosphate
D-Malic acid
Putrescine

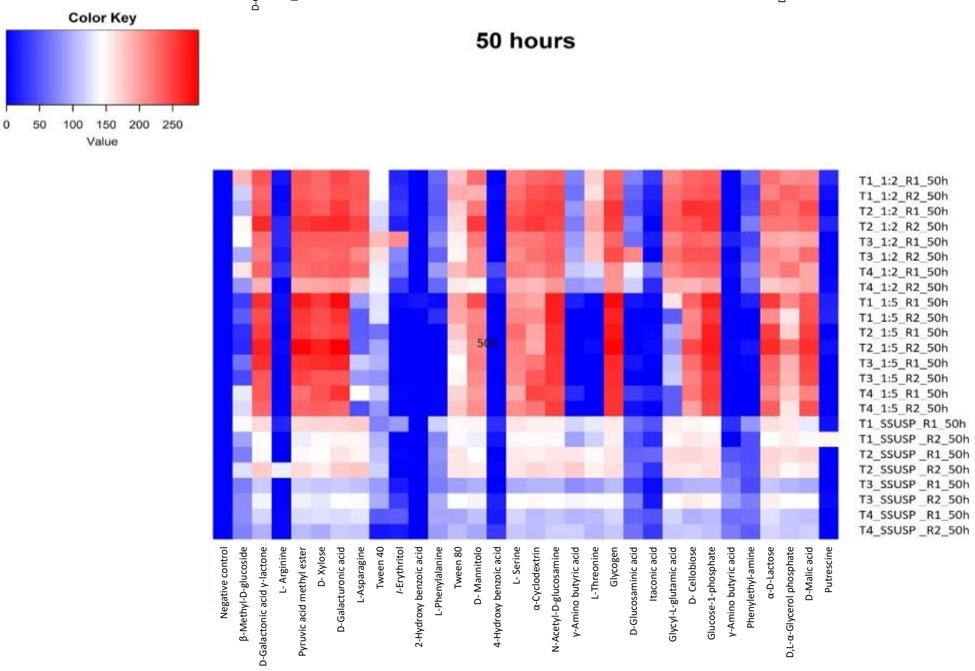
Color Key
0 50 100 150 200 250
Value
50 hours
T1_1:2_R1_50h
T1_1:2_R2_50h
T2_1:2_R1_50h
T2_1:2_R2_50h
T3_1:2_R1_50h
T3_1:2_R2_50h
T4_1:2_R1_50h
T4_1:2_R2_50h
T1_1:5_R1_50h
T1_1:5_R2_50h
T2_1:5_R1_50h
T2_1:5_R2_50h
T3_1:5_R1_50h
T3_1:5_R2_50h
T4_1:5_R1_50h
T4_1:5_R2_50h
T1_SSUSP_R1_50h
T1_SSUSP _R2_50h
T2_SSUSP _R1_50h
T2_SSUSP _R2_50h
T3_SSUSP _R1_50h
T3_SSUSP _R2_50h
T4_SSUSP _R1_50h
T4_SSUSP _R2_50h
Negative control
β-Methyl-D-glucoside
D-Galactonic acid γ-lactone
L- Arginine
Pyruvic acid methyl ester
D- Xylose
D-Galacturonic acid
L-Asparagine
Tween 40
i-Erythritol
2-Hydroxy benzoic acid
L-Phenylalanine
Tween 80
D- Mannitolo
4-Hydroxy benzoic acid
L- Serine
α-Cyclodextrin
N-Acetyl-D-glucosamine
γ-Amino butyric acid
L-Threonine
Glycogen
D-Glucosaminic acid
Itaconic acid
Glycyl-L-glutamic acid
D- Cellobiose
Glucose-1-phosphate
γ-Amino butyric acid
Phenylethyl-amine
α-D-Lactose
D,L-α-Glycerol phosphate
D-Malic acid
Putrescine

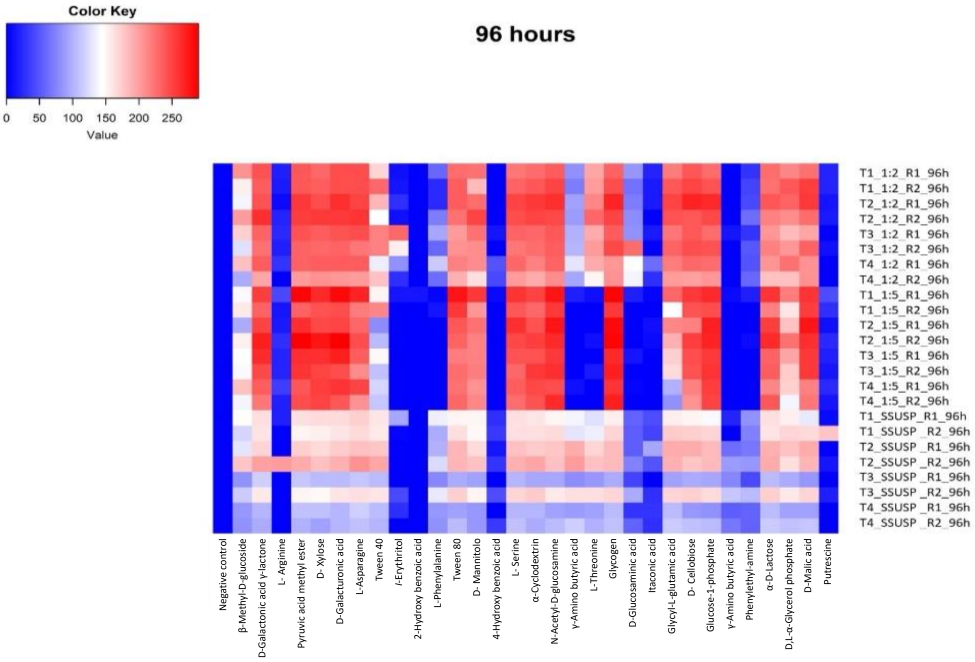
Color Key
0 50 100 150 200 250
Value
96 hours
T1_1:2_R1_96h
T1_1:2_R2_96h
T2_1:2_R1_96h
T2_1:2_R2_96h
T3_1:2_R1_96h
T3_1:2_R2_96h
T4_1:2_R1_96h
T4_1:2_R2_96h
T1_1:5_R1_96h
T1_1:5_R2_96h
T2_1:5_R1_96h
T2_1:5_R2_96h
T3_1:5_R1_96h
T3_1:5_R2_96h
T4_1:5_R1_96h
T4_1:5_R2_96h
T1_SSUSP_R1_96h
T1_SSUSP _R2_96h
T2_SSUSP _R1_96h
T2_SSUSP _R2_96h
T3_SSUSP _R1_96h
T3_SSUSP _R2_96h
T4_SSUSP _R1_96h
T4_SSUSP _R2_96h
Negative control
β-Methyl-D-glucoside
D-Galactonic acid γ-lactone
L- Arginine
Pyruvic acid methyl ester
D- Xylose
D-Galacturonic acid
L-Asparagine
Tween 40
i-Erythritol
2-Hydroxy benzoic acid
L-Phenylalanine
Tween 80
D- Mannitolo
4-Hydroxy benzoic acid
L- Serine
α-Cyclodextrin
N-Acetyl-D-glucosamine
γ-Amino butyric acid
L-Threonine
Glycogen
D-Glucosaminic acid
Itaconic acid
Glycyl-L-glutamic acid
D- Cellobiose
Glucose-1-phosphate
γ-Amino butyric acid
Phenylethyl-amine
α-D-Lactose
D,L-α-Glycerol phosphate
D-Malic acid
Putrescine

Supplementary Fig. S3. Heatmap plots of Metabolic activity value (MAV) in different sources at 20, 50 and 96 hours after incubation of fecal samples stored for a different time (T1, T2, T3 and T4) and analyzed at different sample dilution (SSUSP, 1:2 and 1:5).
